# Medial elbow anatomy: A paradigm shift for UCL injury prevention and management

**DOI:** 10.1002/ca.23322

**Published:** 2019-01-09

**Authors:** Shota Hoshika, Akimoto Nimura, Reiko Yamaguchi, Hisayo Nasu, Kumiko Yamaguchi, Hiroyuki Sugaya, Keiichi Akita

**Affiliations:** ^1^ Department of Clinical Anatomy, Graduate School of Medical and Dental Science Tokyo Medical and Dental University Tokyo Japan; ^2^ Shoulder & Elbow Service Funabashi Orthopaedic Sports Medicine & Joint Center Funabashi Chiba Japan; ^3^ Department of Functional Joint Anatomy, Graduate School of Medical and Dental Sciences Tokyo Medical and Dental University Tokyo Japan

**Keywords:** elbow, collateral ligament, ulnar, anatomy and histology, baseball, rehabilitation

## Abstract

To improve the management outcomes and diagnostic accuracy of the ulnar collateral ligament (UCL) injury, the anatomy of the medial side of the elbow joint is necessary to be understood in terms of the periarticular surroundings rather than the specific ligaments. The aim of this study was to anatomically clarify the medial side of the elbow joint in terms of the tendinous structures and joint capsule. We conducted a descriptive anatomical study of 23 embalmed cadaveric elbows. We macroscopically analyzed the relationship between the flexor pronator muscles (FPMs) and the joint capsule in 10 elbows, histologically analyzed in 6 elbows, and observed the bone morphology through micro computed tomography in 7 elbows. The two tendinous septa (TS) were found: between the pronator teres (PT) and flexor digitorum superficial (FDS) muscles, and between the FDS and flexor carpi ulnaris (FCU) muscles. These two TS are connected to the medial part of the brachialis tendon, deep aponeurosis of the FDS, and FCU to form the tendinous complex, which linked the humeroulnar joint and could not be histologically separated from each other. Moreover, the capsule of the humeroulnar joint under the tendinous complex had attachment on the ST of 7 mm width. The two TS, the brachialis tendon, the deep FDS and FCU aponeuroses, and the joint capsule linked the humeroulnar joint. These anatomical findings could lead to a paradigm shift in the prevention, diagnosis, and treatment of UCL injuries in baseball players. Clin. Anat. 32:379–389, 2019. © 2018 The Authors. *Clinical Anatomy* published by Wiley Periodicals, Inc. on behalf of American Association of Clinical Anatomists.

## INTRODUCTION

Ulnar collateral ligament (UCL) injuries of the elbow joint have become common among overhead throwing athletes, particularly baseball pitchers (Fleisig and Andrews, 2012). Recent studies reported the failure of nonoperative management of UCL injury in overhead athletes (Rettig et al., 2001; Ford et al., 2016; Frangiamore et al., 2017). On the contrary, UCL reconstructions in baseball players have been proven to be an effective procedure for return to play and performance (Erickson et al., 2014; Osbahr et al., 2014; Hodgins et al., 2016). However, the rapidly increasing number of reconstructions is reported in the amateur and adolescent athletes (Jones et al., 2014; Hodgins et al., 2016). The avoidance of unnecessary surgeries associated with long recovery period should be beneficial in specific clinical scenarios (Rebolledo et al., 2017). Therefore, improvement of nonoperative management outcomes and diagnostic accuracy for UCL injury are necessary to avoid surgery.

For the nonoperative management for the UCL injuries, such as the rehabilitation, the anatomic and biomechanical understanding of the medial elbow joint is necessary. The anterior bundle of the UCL has been thought as the primary static stabilizer against valgus stress during throwing motion (Morrey and An, 1985). In addition, the flexor‐pronator muscles (FPMs), including the flexor carpi radialis (FCR), pronator teres (PT), flexor digitorum superficial (FDS), and flexor carpi ulnaris (FCU) muscles, have also been assumed to contribute to dynamic stabilization (An et al., 1981; Sisto et al., 1987; Morrey et al., 1991; Digiovine et al., 1992; Glousman et al., 1992; Davidson et al., 1995; Hamilton et al., 1996; Lin et al., 2007; Udall et al., 2009; Frangiamore et al., 2017). However, the anatomical relationship between static and dynamic stabilizers, or how the FPMs are connected to the anterior bundle, has been rarely discussed (Davidson et al., 1995; Munshi et al., 2004; Frangiamore et al., 2017). Because the word “ligament” anatomically means a simple band connecting bones to bone, and is not the absolute definition, the conventional anatomical analysis based on the specific ligaments might have its limits to understand the anatomical structures for the joint stabilization.

Furthermore, for precise evaluation of UCL injuries in throwing athletes, the magnetic resonance arthrogram is conventionally used to detect undersurface tears of the anterior bundle of the UCL, which has been referred as the T‐sign (Timmerman et al., 1994; Timmerman and Andrews, 1994a). However, the location of the attachment area of the anterior bundle of the UCL on the sublime tubercle (ST) remains controversial (Timmerman and Andrews, 1994a; Cage et al., 1995; Munshi et al., 2004; Dugas et al., 2007). Meanwhile, a previous study reported that the capsule of the anterior elbow joint had substantial attachment width in the sagittal direction, and the cartilage surface did not have the capsular attachment at the tip of the coronoid process (Shimura et al., 2016). However, in the medial elbow joint, the attachment of the underlying capsule and the layered relationship between the UCL and the joint capsule have been rarely analyzed. Therefore, the actual dimension of the anterior bundle, particularly in reference to the capsular attachment on the ST, is still unclear.

Based on the above description, we hypothesized that the anatomical findings based on the surroundings including the tendinous structures and joint capsule, which constantly existed around the joint, could give some clues for the improvement of UCL injury management. The aim of the present study was to anatomically analyze the medial side of the elbow joint in terms of the tendinous structures and joint capsule rather than the specific ligaments.

## MATERIALS AND METHODS

### Donors

We used a total of 24 elbows (11 right and 13 left) from 16 Japanese cadavers (eight male and eight female cadavers; average age at death, 83 years old, range, 49–99 years old) in this study. All cadavers were donated to the department of anatomy of the Tokyo Medical and Dental University. In eight cadavers, both elbows were assigned for the current study. In the remaining eight cadavers, elbows on only one side were assigned. All donors had voluntarily declared that their remains were to be donated as materials for education and study. This voluntary donor system of cadavers is in place throughout Japan, and our study completely complies with the current laws of Japan. We fixed all cadavers in 8% formalin and preserved them in 30% ethanol. The arm was obtained by sectioning the middle of the humerus. The skin and subcutaneous tissues were removed from the arm. In 24 elbows, we excluded one specimen with severe osteophytes from the analyses. A total of 17 elbows were used for macroscopic observations and measurements, including 6 elbows from the same three individuals. A total of 6 elbows were used for histological analysis.

### Macroscopic Observation and Measurements of Capsular Attachments

In 17 elbows, we firstly observed the outer appearance of the FPMs and then removed the superficial fascia of the medial side of the elbow joint. To identify the muscular and tendinous structures of FPMs and nerves, we removed all muscular parts of the palmaris longus (PL), FCR, PT, FDS, and FCU muscles. Then, to reveal the relationship between the tendinous part of the FPMs and surrounding structures, we removed all muscular parts of the FPMs and brachialis tendon. In addition, we detached the tendinous structures and the joint capsule together from the lateral to medial to identify the capsular attachment on the ulna. We measured the dimensions of the capsular attachment and footprints of tendinous structures of the FPMs using a caliper.

### Histological Investigation

We analyzed six elbows histologically. We removed the medial part of the humeroulnar joint, including the medial epicondyle (MEC) and the ST, en bloc using a diamond band pathology saw (EXAKT 312, EXAKT Advanced Technologies GmbH, Norderstedt, Germany). We decalcified the specimen for one week in a solution containing aluminum chloride, hydrochloric acid, and formic acid, as described by Plank and Rychlo (1952). After decalcification, we sectioned the blocks in the axial plane at the level of the ST just distal to the joint (three elbows) and in the oblique coronal plane at the level of the anterior slope of the MEC (three elbows). We dehydrated the blocks with a graded ethanol series. After dehydration, we embedded the tissue blocks in paraffin and serially sectioned them (5 μm thickness) parallel to the sectioned plane. We stained the sections with Masson trichrome.

### Bony Morphology with Micro CT

In 17 elbows for macroscopic observations, we randomly selected seven elbows for observation of the bone morphology. We took three‐dimensional (3D) images using a micro‐computed tomography (CT) scanner (inspeXio SMX‐100CT, Shimadzu, Kyoto, Japan) with application software (VGStudio Max 2.0, Heidelberg, Germany). To identify the relationship between the ST and the tendinous structures of the FPMs, radiopaque markers (1 mm^2^) were placed at 5 mm intervals along the corner of the anterior bases of the tendinous septa (TS), which were described below.

### Statistical Analysis

Statistical analyses were performed with IBM SPSS Statistics 18 software for Windows (IBM Japan Inc.). Analysis of interobserver reliability yielded an interclass correlation coefficient of 0.89–0.96.

## RESULTS

### Identification of Muscular and Tendinous Structures of FPMs in the Elbow Joint

**Figure 1:**
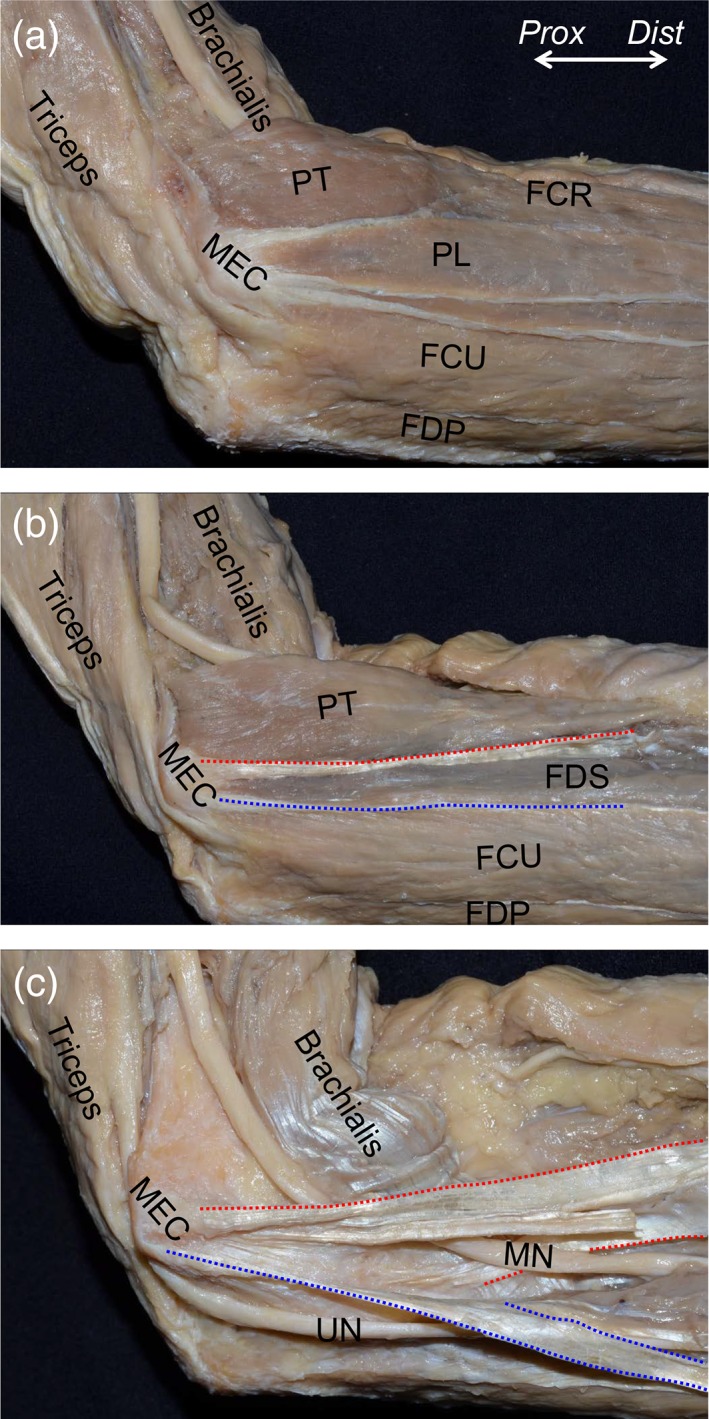
Muscular and tendinous structures of the flexor‐pronator muscles (FPMs). The medial aspect of the left elbow is shown. (**A**) After removal of the superficial fascia, FPMs were shown to form a common muscular origin on the medial supracondylar ridge and the medial epicondyle of the humerus (MEC). (**B**) The palmaris longus (PL) and flexor carpi radialis (FCR) muscles were removed, and the flexor digitorum superficialis (FDS) muscle was exposed. Between the pronator teres (PT) and FDS muscles (red dotted line), and the FDS and flexor carpi ulnaris (FCU) muscles (blue dotted line), the tendinous septa (TS) could be identified. (**C**) After removal of muscular parts of the PT, FDS, and FCU muscles, the median nerve (MN) was shown to penetrate the TS between the PT and FDS muscles (red dotted area), and the ulnar nerve (UN) passed posterior to the TS between the FDS and FCU muscles (blue dotted area). FDP, flexor digitorum profundus; *Dist*, distal; *Prox*, proximal. [Color figure can be viewed at https://wileyonlinelibrary.com]

**Figure 2:**
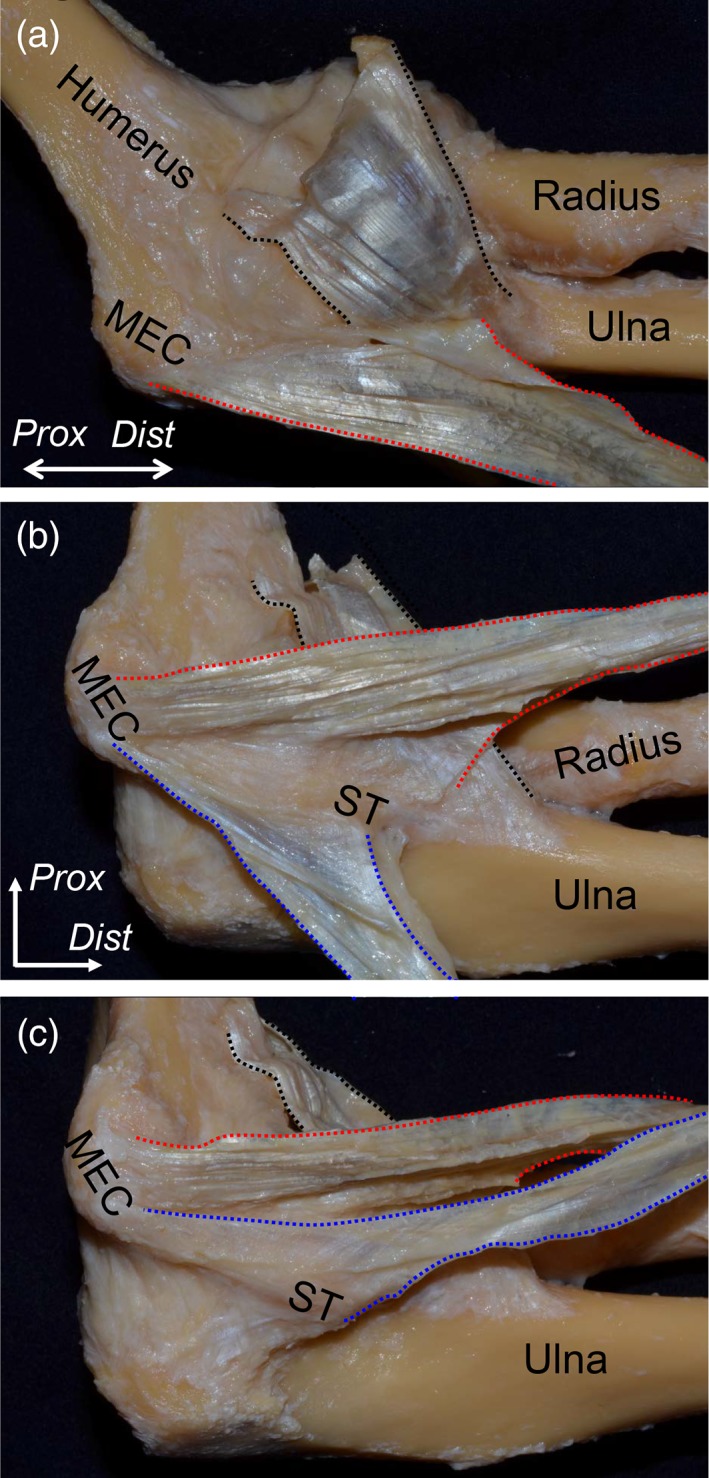
Relationship between the TS and its surrounding structures. All muscular parts of the FPMs and nerves were removed. (**A**) The tendinous part of the brachialis muscle (black dotted area) and the anteromedial aspect of the TS between the PT and FDS muscles (red dotted area) of the left elbow are shown in the extended position. The brachialis tendon was mainly inserted into the coronoid process and partially connected to the base of the TS between the PT and FDS muscles at the sublime tubercle (ST) of the ulna. (**B**) In the flexion position of the elbow, the TS between the PT and FDS muscles was reflected anteriorly. It originated from the anterior slope of the medial epicondyle (MEC), distally extended to the anterior part of the ST, and transitioned into the deep aponeurosis of the FDS muscle over the humeroulnar joint. In addition, the TS between the FDS and FCU muscles was reflected posteriorly (blue dotted area). (**C**) The TS between the FDS and FCU muscles was reflected anteriorly. It originated from the posterior slope of the MEC, distally extended to the posterior part of the ST, and posteriorly transitioned into the deep aponeurosis of the FCU muscle over the humeroulnar joint. *Dist*, distal; *Prox*, proximal. [Color figure can be viewed at https://wileyonlinelibrary.com]

Before observing the muscular parts of FPMs, we removed the superficial fascia of the medial side of the elbow joint. FPMs formed a common muscular origin on the medial supracondylar ridge and the MEC of the humerus (Fig. 1A). After removal of the PL and FCR muscles, the FDS muscle was exposed (Fig. 1B). Between the PT and FDS muscles (red dotted line in Fig. 1B), and the FDS and FCU muscles (blue dotted line in Fig. 1B), two TS could be identified. To understand the deep parts of the TS and nerves, we removed the muscular parts of the PT, FDS, and FCU muscles. The median nerve penetrated the TS between the PT and FDS muscles (red dotted area in Fig. 1C). The ulnar nerve passed posterior to the TS between the FDS and FCU muscles (blue dotted area in Fig. 1C). To reveal the relationship between the TS and surrounding structures, all remaining muscular parts and the median and ulnar nerves were removed. The brachialis tendon is mainly inserted into the coronoid process and partially connected to the base of the TS between the PT and FDS muscles at the ST of the ulna (Fig. 2A). The TS between the PT and FDS muscles originated from the anterior slope of the MEC, distally extended to the anterior part of the ST, reflected to posterior, and transitioned into the deep aponeurosis of the FDS muscle over the humeroulnar joint (Fig. 2B). The TS between the FDS and FCU muscles originated from the posterior slope of the MEC, distally extended to the posterior part of the ST, and posteriorly transitioned into the deep aponeurosis of the FCU muscle over the humeroulnar joint (Fig. 2C). The medial part of the brachialis tendon, the two TS, and the deep FDS and FCU aponeuroses formed the tendinous complex, which could not be separated from each other.

### Ulnar Attachments of the Joint Capsule Under the Tendinous Complex

**Figure 3:**
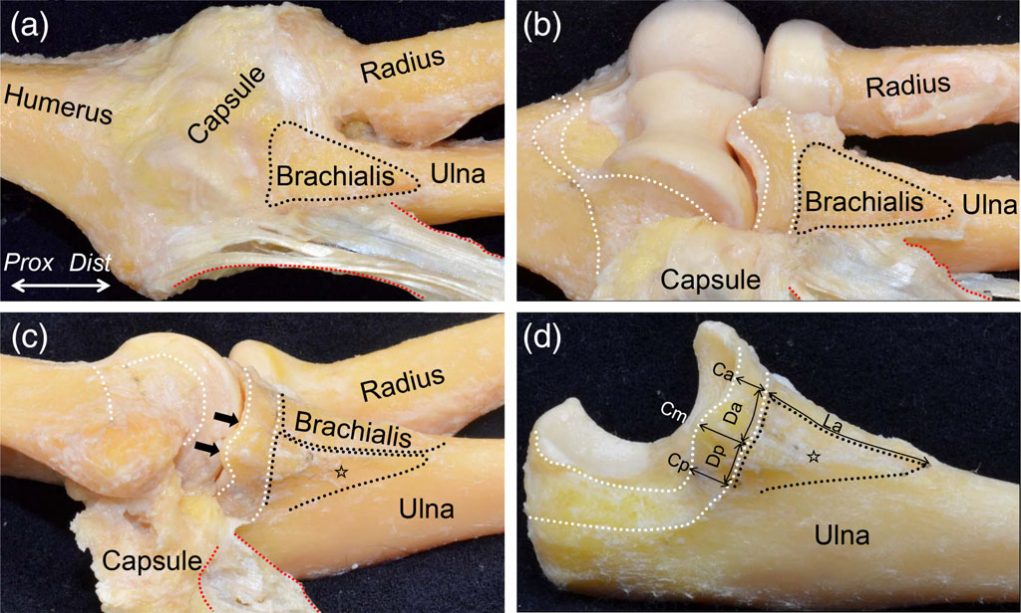
Ulnar attachments of the tendinous complex and the capsule of the elbow joint. (**A**) The appearance after removing the brachialis tendon from Fig. 2 is shown. The ulnar insertion of the brachialis tendon (black dotted area) and the TS between the PT and FDS muscles (red dotted area) are shown. (**B**) The joint capsule was detached from the lateral to the medial margin of the brachialis insertion and medially reflected. The capsular attachments on the bones are shown as white dotted area. (**C**) Furthermore, we detached posteriorly the joint capsule and the tendinous complex containing the two TS and the deep FDS aponeurosis. The ulnar insertion of the tendinous complex is shown as the open star. The widths of the cartilage surface without the capsular attachment were too small to measure (black arrows). (**D**) Finally, the joint capsule and the tendinous complex were detached together from the ulna. The picture indicates measurements of the insertion of the tendinous complex and the attachment of the joint capsule on the ulna. Measured locations and data are shown in Table 1. *Dist*, distal; *Prox*, proximal. [Color figure can be viewed at https://wileyonlinelibrary.com]

To analyze the ulnar attachments of the joint capsule under the tendinous complex, the ulnar insertion of the brachialis tendon was detached from the coronoid process (black dotted area in Fig. 3A), and the medial part connecting to the TS was cut. Then, the TS and the joint capsule were detached and reflected together from the lateral to medial direction (Fig. 3B, C). While the joint capsule of the elbow adjoined with the overlying tendinous complex, the attachment width could be identified at the medial margin of the brachialis insertion and the anterior margin of the ulnar attachments of the TS. At the anterior border of the TS, the width of the capsular attachment was 6.4 ± 0.5 mm (average ± standard deviation, Ca in Fig. 3D). At the tip of the ST, the maximum width of the capsular attachment (7.3 ± 0.6 mm, Cm in Fig. 3D) was found, which was located 3.6 ± 0.5 mm posterior to the anterior border of the TS (Da in Fig. 3D). Based on the macroscopic observation, the widths of the cartilage surface without the capsular attachment were too small to measure (~0–1 mm). The average attachment widths of the tendinous complex and the joint capsule are shown in Table 1.

**Table 1:** Measurement of the Attachments of the Tendinous Complex and Joint Capsule on the Sublime Tubercle

Locations of the measurement	Average and standard deviation (mm)
Capsular attachment on the sublime tubercle	
Width at the anterior border of the tendinous complex (Ca)	6.4 ± 0.5
Width at the posterior border of the tendinous complex (Cp)	6.2 ± 0.4
Maximum width of capsular attachment (Cm)	7.3 ± 0.6
Distance from anterior border of the tendinous complex to the point of maximum width of capsular attachment (Da)	3.6 ± 0.5
Distance from posterior border of the tendinous complex to the point of maximum width of capsular attachment (Dp)	2.8 ± 0.4
Ulnar attachments of the tendinous complex	
Length at the anterior border of the tendinous complex (La)	26.4 ± 1.3

Locations of measurements are demonstrated in Figure 3D.

### Histological Analysis of the Tendinous Complex and Joint Capsule

**Figure 4:**
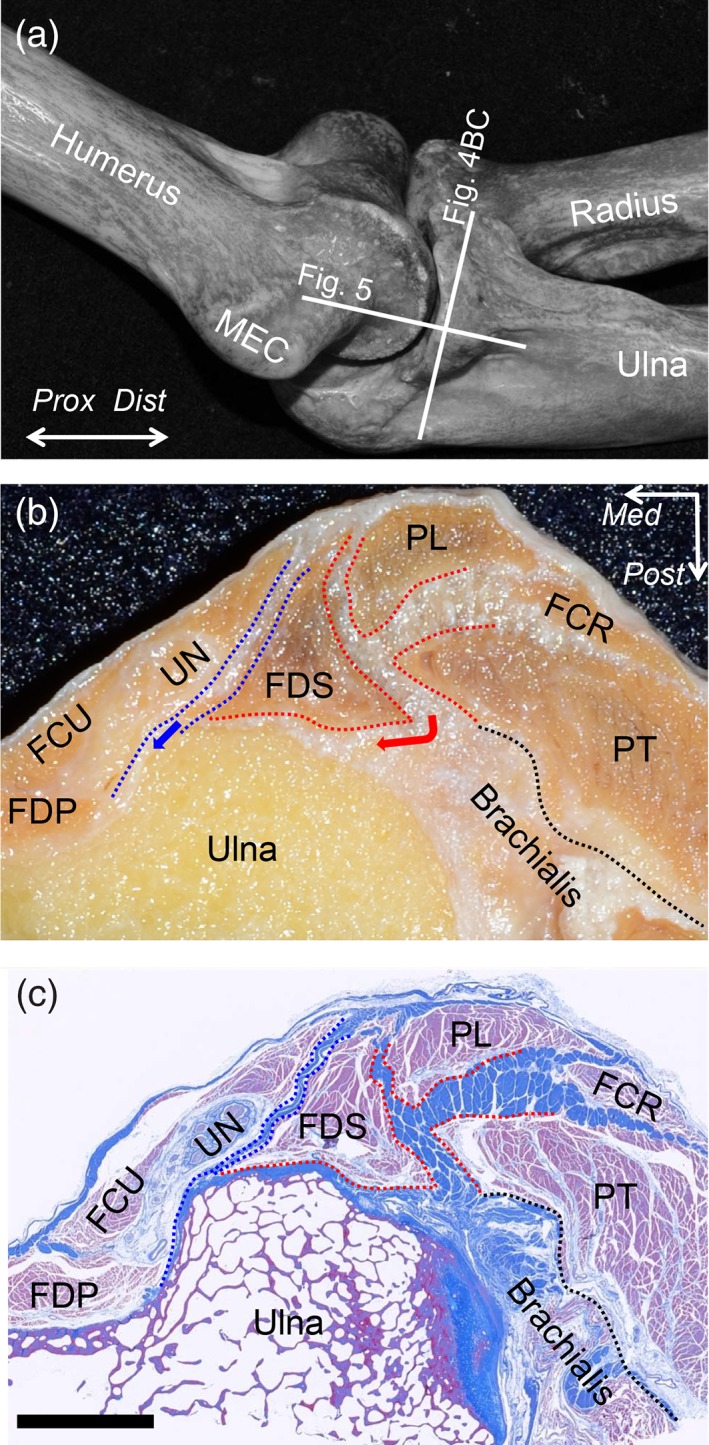
Histological analysis of the axial section at the sublime tubercle. (**A**) Locations of the histological section in Figs. 4 and 5 are indicated as white lines on the bony scheme of the medial aspect of the left elbow. (**B**) The macroscopic view of the axial section at the level of the ST is shown as the white line in **A**. (**C**) Masson's trichrome staining of the section is shown in (**B**). The TS between the PT and FDS muscles was densely stained (red dotted area), connected to the intramuscular tendon of the brachialis muscle (black dotted line), and transitioned into the deep FDS aponeurosis (red arrow). The TS between the FDS and FCU muscles was also identified (blue dotted area) and continued into the deep FCU aponeurosis (blue arrow). These two TS, the intramuscular tendon of the brachialis muscle, and the deep aponeuroses of the FDS and FCU muscles were connected and formed the tendinous complex. FCR, flexor carpi radialis; FCU, flexor carpi ulnaris; FDP, flexor digitrum profundus; FDS, flexor digitrum superficialis; MEC, medial epicondyle; PT, pronator teres; PL, palmaris longs; UN, Ulnar nerve; *Dist*, distal; *Prox*, proximal; *Med,* medial; *Post*, posterior, Scale bar, 5 mm [Color figure can be viewed at https://wileyonlinelibrary.com]

To reveal the relationship between the tendinous complex and its surrounding structures, we histologically analyzed the axial and oblique coronal sections of the medial side of the humeroulnar joint. In the axial section at the level of the ST just distal to the joint, we could identify the TS between the PT and FDS muscles as a densely stained structure (red dotted area in Fig. 4B, C). The TS between the PT and FDS muscles was connected to the intramuscular tendon of the brachialis muscle (black dotted line in Fig. 4B) and transitioned into the deep aponeurosis of the FDS muscle. We also identified the TS between the FDS and FCU muscles (blue dotted area in Fig. 4B, C), which was less developed than the TS between the PT and FDS muscles, and continued into the deep aponeurosis of the FCU muscle (Fig. 4B,C).

**Figure 5:**
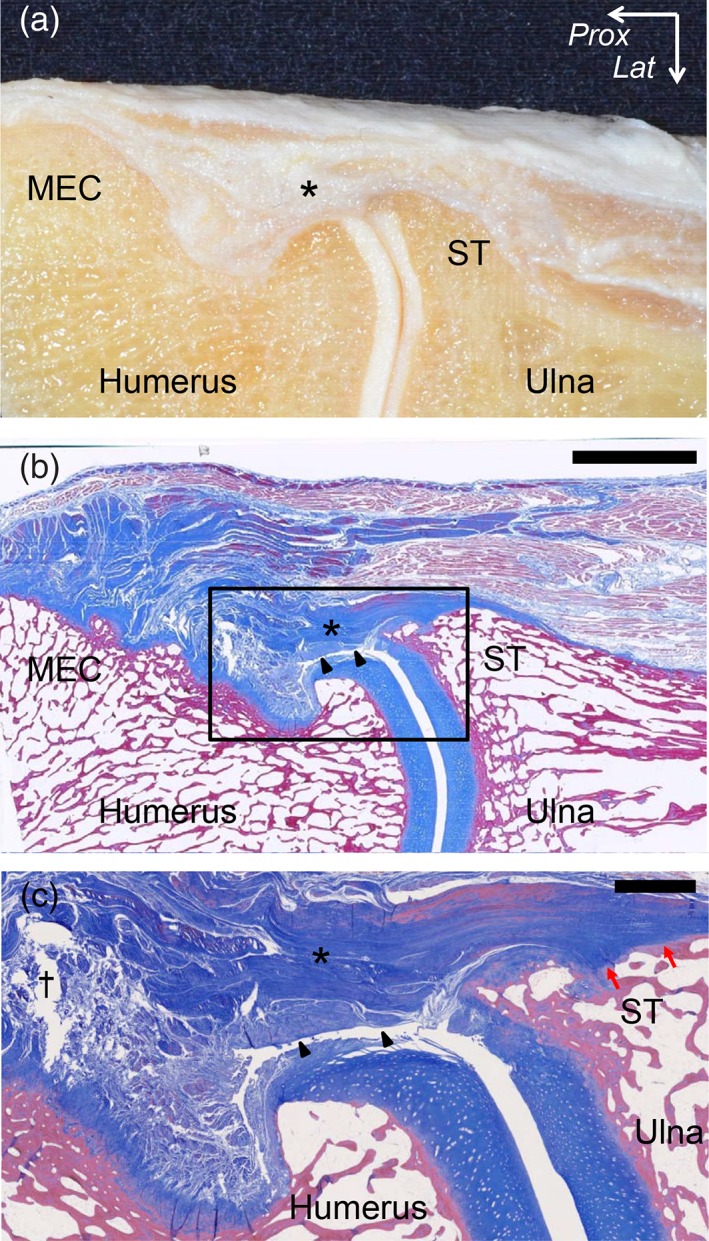
Histological analysis of the oblique coronal section at the TS between the PT and FDS muscles. (**A**) The macroscopic view of the oblique coronal section at the level of the TS between the PT and FDS muscles is shown in Fig. 4A. (**B**) Masson's trichrome staining of the section is shown in **A**. The joint capsule (black arrowheads) was identified under the TS (asterisk). (**C**) Magnified image of the part is shown as black square in (**B**). Proximal to the humeroulnar joint, the capsule reflected to form a synovial cavity (black cross) with a distinct margin from the TS. Distal to the humeroulnar joint, the capsule intermingled with the TS and attached together to the sublime tubercle (ST) with a fibrocartilage (red arrows). *Lat*, lateral; *Prox*, proximal. Scale bar, 5 mm in **B**, 1 mm in **C**. [Color figure can be viewed at https://wileyonlinelibrary.com]

In the oblique coronal section at the level of the anterior slope of the MEC of the humerus (Fig. 4A), the joint capsule (black arrowheads in Fig. 5B) was found under the TS. Proximal to the humeroulnar joint, the capsule reflected to form a synovial cavity with a distinct margin from the TS (black cross in Fig. 5C). However, distal to the joint, the capsule intermingled with the TS and attached together to the ST with a fibrocartilage (red arrows in Fig. 5C).

### Micro‐CT Evaluation of the Relationship Between the Bone Morphology and TS

**Figure 6:**
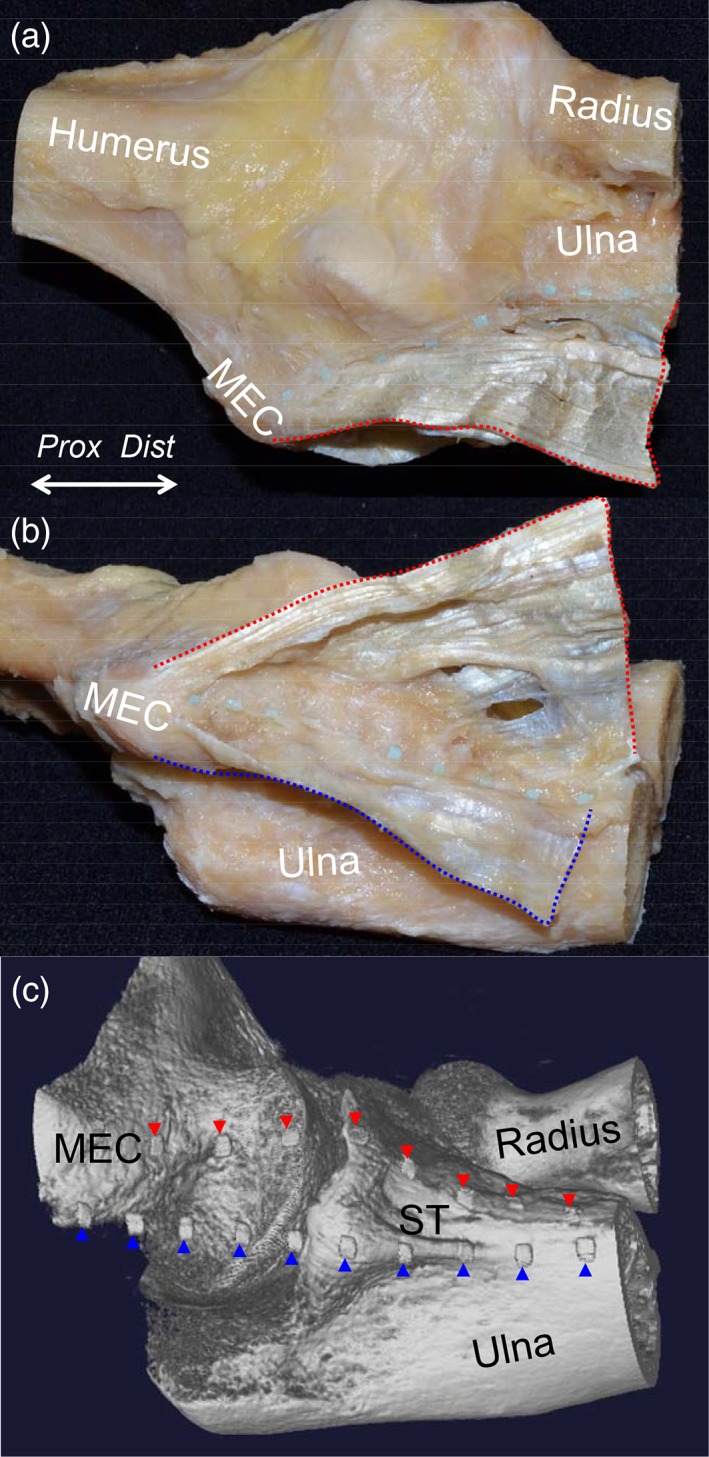
Relationship between the TS and the bone morphology, showing the cadaveric sample of the elbow with the tendinous complex of the left elbow. (**A**) The radiopaque markers were set along the anterior base of the TS between the PT and FDS muscles (red dotted area). (**B**) The radiopaque markers were set along the anterior base of the TS between the FDS and FCU muscles (blue dotted area). (**C**) 3D imaging using micro‐CT of the sample with the radiopaque markers. The markers for the TS between the PT and FDS muscles are indicated as red arrowheads and those for the TS between the FDS and FCU muscles are indicated as blue arrowheads. MEC, medial epicondyle; ST, sublime tubercle; *Dist*, distal; *Prox*, proximal. [Color figure can be viewed at https://wileyonlinelibrary.com]

To observe the relationship between the morphology of the bones and the location of the TS, we took 3D images of the elbows, in which we put radiopaque markers on the anterior margin of the two TS (Fig. 6A, B). The anterior border of the TS between the PT and FDS muscles corresponded to the anterodistal slope of the MEC and the anterior base of the ST (Fig. 6C). The anterior border of the TS between the FDS and FCU muscles corresponded to the posterodistal slope of the MEC and the posterior base of the ST. These anterior borders of the two TS mingled with each other at the distal base of the ST.

## DISCUSSION

The present study revealed two TS found between the PT and FDS muscles and between the FDS and FCU muscles. Moreover, these two TS were connected to the medial part of the brachialis tendon and the deep aponeuroses of the FDS and FCU muscles, forming the tendinous complex. This complex linked the humeroulnar joint, and histological separation was not possible. Furthermore, the present study showed that the capsule of the humeroulnar joint under the tendinous complex had attachment on the ST just distal to the joint with approximately 7 mm width.

Previously, anatomical studies reported that the FPMs had tendinous fibers merging in close proximity to the anterior bundle of the UCL (Davidson et al., 1995; Munshi et al., 2004; Frangiamore et al., 2017). Meanwhile, Otoshi et al. (2014) described that the PT, FCR, PL, and FDS muscles were combined together to form the common tendon at the proximal origin, and the common tendon was attached to the anterior bundle of the UCL. Otoshi et al. (2014) additionally indicated the discrepancy as follows: the common tendon and the anterior bundle of the UCL could be macroscopically clearly distinguished, but their histological morphologies were quite similar.

Originally, the word “ligament” has been thought to consist of fibers, which are arranged in parallel like tendons, were less regularly arranged than those of tendons, and more regularly arranged than those of aponeuroses (Pawlina and Ross, 2016). In other words, the “ligament” anatomically means a simple band connecting bones to bones, and is not the absolute definition, because the differences between the aponeuroses, ligaments, and tendons are not clear. As a result, there have been possibilities that any periarticular structures connecting bones to bones could be referred as the “ligament” regardless of histological definitions. Therefore, the conventional anatomical analysis based on the specific ligaments might have its limits to understand the anatomical structures for the joint stabilization. Thus, we conducted anatomical observations based on the periarticular surrounding, including the tendinous structures and joint capsule, which constantly existed around the joint. At the medial side of the elbow joint, we identified the tendinous complex, which consisted of two TS, the medial part of the brachialis tendon, and the deep FDS and FCU aponeuroses. This complex linked the humeroulnar joint. The thick, collagenous part of the tendinous complex, which corresponded to the base of the TS between the PT and FDS muscles, and the deep FDS aponeurosis could be considered as the anterior bundle of the UCL, as previously understood. In other words, if the collagenous parts were to be artificially removed from the tendinous complex, the anterior bundle of the UCL could be identified as described in previous textbooks and articles.

Ulnar neuropathy is a common elbow injury in throwing athletes (Cain Jr et al., 2003) and is often caused by compression of the ulnar nerve at the elbow (Bozentka, 1998). Therefore, identifying the correlation between the tendinous structures of the medial elbow and the ulnar nerve is important. Previous anatomical studies have reported that the ulnar nerve traversed the upper arm from the anterior to the posterior compartment at the arcade of Struthers to enter the cubital tunnel, the floor of which consisted of the olecranon, medial joint capsule, posterior bundle of the UCL, and the transverse ligament (e.g., Cooper's ligament) (Morrey and An, 1985; Granger et al., 2017). Previous reports have also described that the compression of the ulnar nerve may occur at several sites along this path, such as Osborne's ligament (Bozentka, 1998; Granger et al., 2017), the brachial ligament (Wehrli and Oberlin, 2005), the anconeus epitrochlearis (Grewal et al., 2018), and the subanconeus muscle, of which the highest concentration of fibers was in the joint capsule near the groove of the ulnar nerve (Tubbs et al., 2006). In the current study, we identified the TS between the FDS and FCU, in addition to the TS between the PT and FDS muscles. Given that the TS between the FDS and FCU posteriorly transitioned into the deep aponeurosis of the FCU muscle, this might be related to the pathology of ulnar nerve compression.

With regard to the joint capsule at the medial side of the elbow, Munshi et al. (2004) reported that the anterior bundle of the UCL was distinct from the surrounding synovium and subsynovial fibrous tissues. On the contrary, Timmerman and Andrews (1994b) described that the distinct ligamentous bundles within the joint capsule corresponded to the anterior and posterior bundles of the UCL, which was similar to the inferior glenohumeral ligament complex of the shoulder (O'Brien SJ et al., 1990). Although these appeared controversial, the findings in the current study could explain the discrepancy between claims. Proximal to the humeroulnar joint, the joint capsule was histologically separated from the tendinous complex and reflected to form a synovial cavity. Conversely, distal to the joint, the capsule merged with the tendinous complex and attached together. Thus, the layered relationship between the tendinous complex and the humeroulnar joint capsule was not consistent, but rather varied according to locations.

There have been inconsistencies in the anatomical knowledge of the distal attachment of the anterior bundle of the UCL. Previous studies have variously described that the anterior bundle insertion was 1–4 mm distal to the ulnar articular margin (Timmerman et al., 1994; Cage et al., 1995; Munshi et al., 2004; Dugas et al., 2007). However, previous studies had not identified the width of the capsular attachment, which should underlie the anterior bundle. Meanwhile, at the lateral side of the proximal ulna, Shimura et al. (2016) previously showed that there were no 6‐mm attachment on the cartilage surface of the coronoid tip and 12 mm length attachment of the joint capsule at the lateral side of the coronoid process. In the current study, we found the capsular attachment on the ST with approximately 7 mm width, which was wider than previously thought. The tendinous complex also inserted into the ST more distal to the capsular attachment. In addition, the widths of the cartilage surface without the capsular attachment could not be measured. Based on the current study, prior studies might have measured the mixed construct of the joint capsule and the tendinous complex as the attachment of the anterior bundle.

Differently from the specific ligaments, the tendinous structures and joint capsule of the medial side of the elbow joint can be interpreted as follows. The medial side of the elbow joint was superficially linked by the tendinous complex, which consisted of the two TS, the medial part of the brachialis tendon, and the deep FDS and FCU aponeuroses. In addition, the joint was deeply linked by the joint capsule, which is underlying and distally intermingled with the tendinous complex. What was previously known as the anterior bundle of the UCL could be interpreted as the part of the tendinous complex and joint capsule that would remain if such collagenous parts were grossly cut out of the tendinous complex.

**Figure 7:**
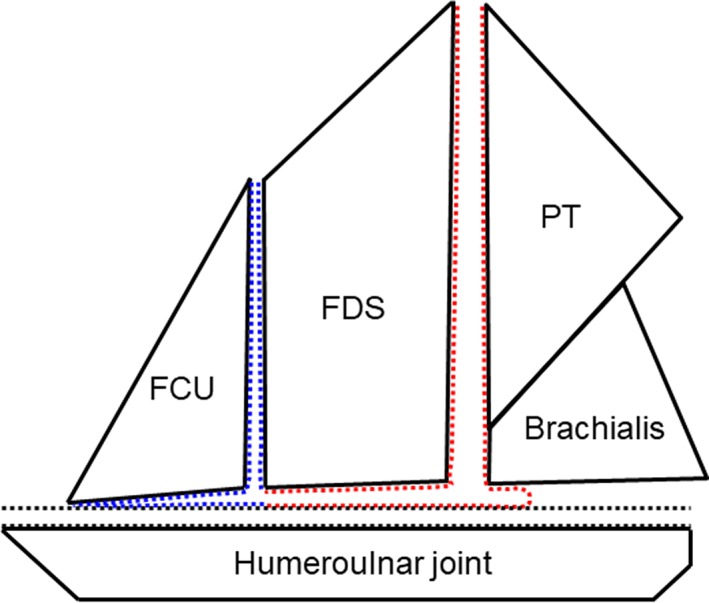
Schematic illustration for the interpretation of the tendinous complex in the medial elbow. The pronator teres (PT), flexor digitorum superficial (FDS), flexor carpi ulnaris (FCU), and brachialis muscles are speculated to work together and transmit the muscular power to the humeroulnar joint via the tendinous complex, which consists of the tendinous septa (TS) between the PT and FDS muscles and the deep FDS aponeurosis (red dotted area), the TS between the FDS and FCU muscles and the deep FCU aponeurosis (blue dotted area), and the joint capsule (black dotted area). The tendinous complex can be assumed to link the medial elbow joint as if the sail transmitted the wind power to the ship. [Color figure can be viewed at https://wileyonlinelibrary.com]

The results of the current study have three clinical implications. First, we were able to improve the understanding of the contribution of the FPMs as dynamic stabilizer in the medial elbow. Some studies using electromyography demonstrated that the PT muscle was activated during the late cocking and acceleration phases (Sisto et al., 1987; Digiovine et al., 1992). Other biomechanical studies demonstrated that the FCU and FDS muscles were the biggest contributor among the FPMs (An et al., 1981; Park and Ahmad, 2004; Lin et al., 2007; Udall et al., 2009). Moreover, other studies showed that the brachialis muscle as well as the FPMs contributed to valgus stability (An et al., 1981; Morrey et al., 1991; Seiber et al., 2009). Based on the current study, the controversy on the contribution of the muscles to stabilize the medial elbow could be explained as follows: The PT, FDS, FCU, and brachialis muscles are speculated not to independently work, but to work together and transmit the muscular power to the humeroulnar joint via the tendinous complex, as if the sail transmitted the wind power to the ship (Fig. 7). The Thrower's Ten, a popular preventive arm care program, incorporates multiple strengthening exercises for the shoulder, elbow, and wrist, but fails to address the finger flexors (Wilk et al., 2002; Fleisig et al., 2015). Taking these anatomical concepts into consideration, the arm care programs for throwing athletes should be reconsidered by adding exercises for the fingers, particularly the FDS muscle.

Second, the “T‐sign,” which indicated the contrast leakage around the detachment of the UCL from the ST (Timmerman et al., 1994), has been important to facilitate magnetic resonance imaging (MRI) diagnosis of partial undersurface tears. In terms of the attachment widths of the tendinous complex and capsule at ST, we could classify the “T‐sign” into three types: type 1 involving less than 1 mm as normal variant, type 2 involving 1–7 mm as capsular detachment, and type 3 involving greater than 7 mm as detachment of the tendinous complex. Thus, based on the anatomical knowledge in the current study, the “T‐sign” could be qualitatively understood according to the detaching locations and structures including the cartilage surface, joint capsule, and the tendinous complex.

Finally, the findings of this study may be useful for improving the outcomes of UCL surgery. Although UCL reconstruction remains the gold standard for treating UCL injuries, there is growing interest in UCL repair with and without augmentation, which has the potential of reducing the time to recovery and the athlete's return to sport performance (Rebolledo et al., 2017; Clark et al., 2018). Historically, UCL repair has obtained good results for selected younger patients (Azar et al., 2000). In addition, recent reports have shown favorable results for repair with augmentation, such as ligament plication, suture repair with bone anchors, or tape augmentation (Savoie et al., 2008; Mackay et al., 2015). Several reports showed that UCL repair with suture tape augmentation had the advantage of a quicker recovery time, decreased soft tissue damage, and better stability, compared with the repair and reconstruction technique (Dugas et al., 2016; Clark et al., 2018). However, a treatment decision for patients should include a consideration of the tissue quality of the UCL (Dugas et al., 2016). Therefore, a precise diagnosis is necessary to select the appropriate patients and improve the outcomes. The results of the present study could be used to diagnose UCL injury precisely and to select patients for non‐reconstructive management. Moreover, our anatomical findings could help to decrease soft tissue disruption, including the tendinous complex and joint capsule, and facilitate a more steady recovery after UCL surgery.

The current study has several limitations. First, this was a purely anatomical study limited to healthy samples. Second, all investigated cadavers were from elderly patients, and this generation was not matched to a peak age of throwing athletes. Given these limitations, the anatomical findings in the current study could serve as a starting point for future research on the muscular contribution of the dynamic stabilizer in the medial elbow against valgus stress during throwing motion using the biomechanical study, and the pathology of UCL injuries using the clinical study. Third, six elbows used in the macroscopic observations and measurements were harvested from the same individuals.

In this study, we found that the tendinous complex, which consisted of the two TS, the medial part of the brachialis tendon, and the deep FDS and FCU aponeuroses, linked the humeroulnar joint. In addition, the capsule of the humeroulnar joint under the tendinous complex had attachment on the ST just distal to the joint with approximately 7 mm width. Our findings suggest that the anterior bundle of the UCL should be reconsidered based on the tendinous complex and joint capsule.

### ACKOWLEDGEMENTS

We would like to acknowledge and thank the anonymous individuals who generously donated their bodies so that this study could be performed. We would like to thank Editage (http://www.editage.jp) for English language editing. This study was partly supported by a grant from JA Kyosai Research Institute (Agricultural Cooperative Insurance Research Institute).

## CONFLICT OF INTEREST

The authors declare that they have no conflicts of interest.
